# E7820, an anti-cancer sulfonamide, degrades RBM39 in patients with splicing factor mutant myeloid malignancies: a phase II clinical trial

**DOI:** 10.1038/s41375-023-02050-4

**Published:** 2023-10-09

**Authors:** Jan Philipp Bewersdorf, Maximilian Stahl, Justin Taylor, Xiaoli Mi, Namrata Sonia Chandhok, Justin Watts, Andriy Derkach, Mateusz Wysocki, Sydney X. Lu, Jessie Bourcier, Simon J. Hogg, Jahan Rahman, Sana Chaudhry, Tulasigeri M. Totiger, Omar Abdel-Wahab, Eytan M. Stein

**Affiliations:** 1 Department of Medicine; Leukemia Service, Memorial Sloan Kettering Cancer Center, New York, NY USA; 2 Department of Medical Oncology, Dana-Farber Cancer Institute, Boston, MA USA; 3 Leukemia Program, Department of Medicine, University of Miami, Sylvester Comprehensive Cancer Center, Miami, FL USA; 4 Department of Biostatistics and Epidemiology, Memorial Sloan Kettering Cancer Center, New York, NY USA; 5 Myeloma Service, Department of Medicine, Memorial Sloan Kettering Cancer Center, New York, NY USA; 6 Molecular Pharmacology Program, Sloan Kettering Institute, Memorial Sloan Kettering Cancer Center, New York, NY USA

**Keywords:** Translational research, Acute myeloid leukaemia

Treatment options for relapsed and/or refractory (R/R) MDS and AML are limited. Mutations in genes encoding RNA splicing factors are encountered frequently in patients with AML and in up to 60% of MDS patients [1, 2]. As splicing factor mutations are often mutually exclusive, splicing factor-mutant cells could be dependent on residual wild-type splicing for survival [3, 4]. Targeting residual splicing function could therefore lead to synthetic lethality and constitute a potent therapeutic approach to splicing factor-mutant AML/MDS [5].

RBM39 is a splicing factor essential for survival of AML cells with splicing factor mutations [6, 7]. The anti-cancer sulfonamide E7820 degrades RBM39 and causes global disruption of splicing in preclinical models [6, 8]. A phase I study of solid tumor patients established the recommended phase II dose of E7820 at 100 mg/d with thrombocytopenia, neutropenia, and elevated liver enzymes constituting dose-limiting adverse events [9]. However, there has never been any evaluation of the ability of this class of drugs to degrade RBM39 in patients in vivo.

We conducted an investigator-initiated phase II trial (NCT05024994) of E7820 in patients with R/R splicing factor-mutant AML, MDS, or CMML. Supplementary Table 1 includes key inclusion and exclusion criteria. Patients received 100 mg of E7820 daily during 28-day cycles until relapse, disease progression, development of unacceptable toxicity, allogeneic hematopoietic stem cell transplant, or death.

The study protocol was developed by the authors in collaboration with Eisai Pharmaceuticals and was approved by our Institutional Review Boards. All patients provided informed consent. The study was conducted in accordance with the Declaration of Helsinki.

The primary objective was to evaluate efficacy of E7820 as measured by overall response rate (ORR) within 6 cycles of therapy. The ORR was defined as a composite of CR + CRh per 2017 ELN response criteria for AML patients and as CR + PR for MDS and CMML per the International Working Group 2006 criteria for MDS and 2015 criteria for CMML [10–12]. We also assessed drug effects on RBM39 protein level, global splicing events, changes in variant allele fraction (VAF) of splicing factor mutations, and DCAF15 mRNA levels and their correlation with clinical responses as exploratory, correlative endpoints (Supplementary Methods). Toxicities were tabulated and graded according to the Common Terminology Criteria for Adverse Events Version 5 (CTCAE-5). Response assessment with bone marrow (BM) biopsies was performed at the end of cycle 1 and every 2 cycles thereafter.

This study used an optimal Simon two-stage design. In the absence of an effective salvage therapy for patients with HMA failure and ORR of <10% with intensive chemotherapy or lower-intensity therapy, we used a null unpromising ORR of 10% and a promising rate of 30% to inform the sample size calculation. Per the study design 12 patients were enrolled in the first stage of the study. If no more than one patient achieved a response, the study was planned to close due to lack of efficacy; otherwise, an additional 23 patients were planned to be accrued. As none of the first 12 patients enrolled achieved an objective response, the study was closed for futility.

Twelve patients were treated (7 AML, 5 MDS) with a median age of 77 years (range 71–85). Patients had received a median of 3 lines of prior therapy (range 1–6). Mutations in *SF3B1, SRSF2, U2AF1*, and *ZRSR2* were present in 6 (50%), 5 (42%), 3 (25%), and 1 (8%) patient, respectively (Supplementary Fig. 1). Baseline patient and disease characteristics are provided in Table 1. At data cut-off (2/28/2023), the median duration of treatment with E7820 was 2.5 cycles (range 1–12 cycles) with one patient continuing treatment.

**Table 1:** Baseline patient and disease characteristics.

Variable	Number of patients
Male sex (%)	8 (67%)
Age (median; R)	77 (71–85)
AML (per ELN 2017 classification) [11]	7 (58%)
-de novo AML	1 (8%)
-AML-MRC	6 (50%)
-ELN – adverse risk	7 (58%)
MDS	5 (42%)
-IPSS-R (median; R)	3.5 (1–6.5)
BM blast (median; R)	23.5% (0–55%)
Lines of prior treatment (median; R)	3 (1–6)
-Intensive chemotherapy	0
-HMA/venetoclax	8 (67%)
-HMA monotherapy	5 (42%)
Prior allo-HCT	1 (10%)
Karyotype at enrollment	
-Normal	5 (42%)
-Complex	2 (17%)
-Monosomy 7	2 (17%)
-isolated del(20q)	1 (8%)
-Trisomy 13	2 (17%)
Molecular characteristics at enrollment	
-*SF3B1* (K700E x3; K666N x2; R625G)	6 (50%)
-*SRSF2* (P95R x2; P95H x2; P95L)	5 (42%)
-*ZRSR2* (C187Y)	1 (8%)
-*U2AF1* (Q157P x2; S34F)	3 (25%)
-*RUNX1*	4 (33%)
-RAS pathway (*NRAS, KRAS, PTPN11, NF1*)	5 (42%)
-*ASXL1*	2 (17%)
-*TP53*	0

Among the first 12 patients enrolled, no patient met the primary endpoint leading to study termination for futility. Two patients did not have a repeat BM assessment and were classified as not having achieved a response per study protocol. One patient achieved a transient marrow complete remission (mCR) without hematologic improvement (blast count reduction from 8 to 3%) but had an increase in blast count at subsequent BM assessment 2 cycles later. All other patients had stable or progressive disease as their best response. Serial mutational analysis revealed an overall modest decrease of 4.9% in splicing factor mutation allele frequency (Supplementary Fig. 2).

At a median duration of follow up of 13.1 months, the median overall survival (OS) from time of E7820 initiation to death was 3.8 months (95% confidence interval: 1.5 months – not reached; Supplementary Fig. 3).

The safety profile of E7820 was in line with prior solid tumor studies [9]. Diarrhea and cough were the most common non-hematologic adverse events (AEs) occurring in six (50.0%) and four patients (33.3%), respectively. Anemia and neutropenia were the most common hematologic AEs (16.7% each). Supplementary Table 3 shows AEs that occurred in more than one patient independent of association to E7820 and grading.

Grade 3 or higher adverse events occurred in 10 patients (83.3%) and were classified as possibly treatment-associated in 4 patients (33%). There were two grade 5 events (multi-organ failure and cardiac arrest), which were unrelated to treatment.

There were 21 serious AEs that occurred in 8 patients (66.7%); three of which were classified as treatment-associated (1 case each of grade 3 AST elevation, grade 2 ALT elevation, and grade 4 neutropenia). The transaminase elevations observed in a single patient were transient and resolved with holding of E7820.

Western blotting of peripheral blood (PB) mononuclear cells (MNCs) pre-treatment and on-treatment revealed >50% RBM39 degradation at cycle 2 day 1 of therapy (Fig. 1A and Supplementary Table 3). To evaluate the functional impact of E7820 on splicing, we collected PB and BM MNCs from patients (Supplementary Table 4) and leukemia cell lines with mutations in *SF3B1, SRSF2*, and *U2AF1* following in vitro treatment with E7820 at doses (1 μM treatment for 24 h) previously identified as achieving >90% RBM39 degradation and having anti-cancer effect (Fig. 1B) [13, 14]. DCAF15 expression from RNA-seq from patient PB and BM revealed clear DCAF15 mRNA expression at all time points (Supplementary Fig. 4). Evaluation of global gene expression by principle component analyses revealed that K562 cells, NKM1 cells, and patient samples clustered distinctly from one another regardless of splicing factor genotype or drug treatment (Supplementary Fig. 5).

**Fig. 1:**
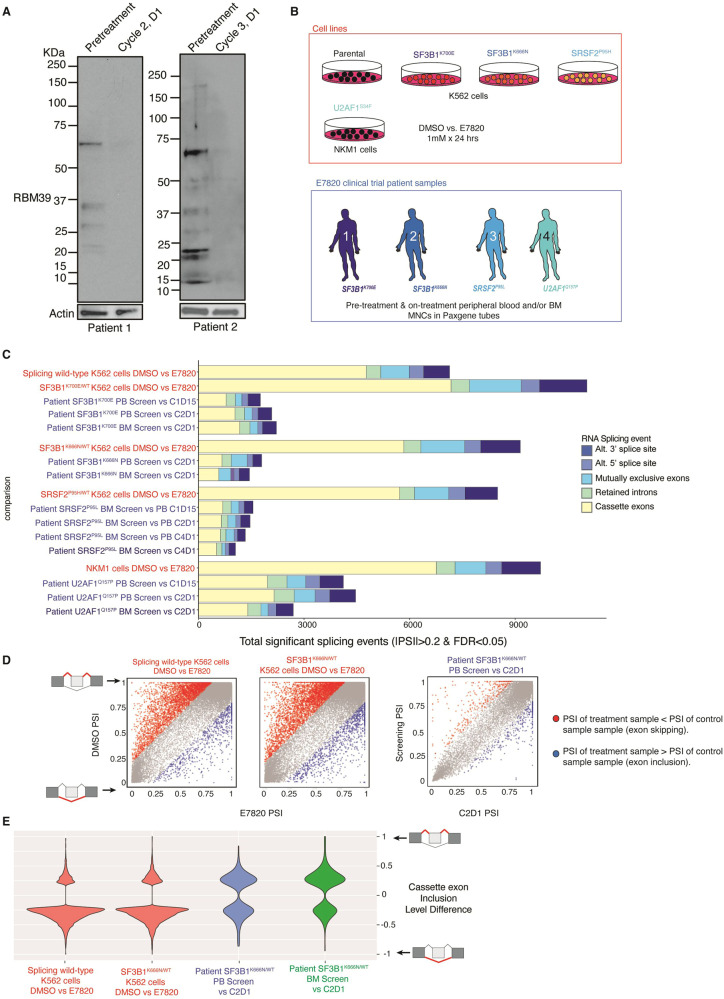
Evaluation of RBM39 degradation and genome-wide changes in RNA splicing upon E7820 treatment in patients as well as preclinical AML models. **A** Full-length Western blot of RBM39 protein in peripheral blood mononuclear cells from two patients at pre- and on-treatment with E7820 (patient details are in Supplementary Table 2). **B** Schema of sample collection for RNA sequencing (RNA-seq). K562 cells with or without knockin of *SF3B1*^K700E^, *SF3B1*^K666N^, and *SRSF2*^P95H^ mutations as well as NKM1 cells (which harbor a *U2AF1* mutation) were treated with 1 μM of E7820 for 24 h and then subjected to RNA-seq. In parallel, RNA from peripheral blood and bone marrow mononuclear cells (PB MNCs and BM MNCs, respectively) from four patients with the indicated RNA splicing factor mutation at screening and on treatment with E7820 in the trial were sequenced in parallel with the cell lines. Baseline patient, clinical, and disease characteristics for these patients is provided in Supplementary Table 4. **C** Enumeration of statistically significant alternative splicing events (defined as absolute value of percent spliced-in (PSI) > 0.2 and FDR < 0.05) on E7820 treatment versus DMSO/pre-treatment in cell lines (shown in red font) or patient samples (blue font). Distinct categories of RNA splicing events are displayed. **D** Scatter plots of differentially spliced cassette exons in splicing factor wild-type K562 cells (left), SF3B1 cells with knockin of the K666N mutation (middle), and the peripheral blood of a patient with *SF3B1*^K666N^ mutation (right) in absence of E7820 (*y*-axis) or presence of E7820 (*x*-axis; cycle 2 day 1 (C2D1) sample for the patient). Red dots, cassette exon events with lower PSI in the treatment sample than in the control sample (exon skipping). Blue dots, cassette exon events with greater PSI in the treatment sample than in the control sample (exon inclusion). As shown, E7820 resulted in cassette exon skipping which was far greater in abundance in the cell lines than the patient sample. **E** Violin plots of the distribution of cassette exon inclusion level difference in cell lines and patient samples on E7820 treatment compared to DMSO/pre-treatment. Each comparison is listed on the *x*-axis and is shown in peripheral blood (PB) and bone marrow (BM) at various timepoints on treatment for the patient.

E7820 induced massive global changes in alternative splicing, which was more pronounced in cell lines bearing splicing factor mutations compared to the parental wild-type splicing cells (Fig. 1C). Similar changes in splicing were seen in PB and BM MNCs from patients treated with E7820 as early as cycle 1 day 15. However, the number of statistically significant splicing events induced by E7820 treatment, was on average ~80% less than those seen in cell lines (Fig. 1C, D). Overlap in individual E7820-dysregulated RNA splicing events among mutant cell lines and patient samples was modest (Fig. 1E and Supplementary Fig. 6A). E7820 resulted in mis-splicing of a number of transcripts encoding proteins required for DNA repair and cell cycle in cell lines, which were not induced in patient tissues (Supplementary Fig. 6B, C). Although such results could be due to distinct gene expression in cell lines compared to primary patient MNCs, similar findings were seen across multiple patient samples and cell lines (Supplementary Fig. 1C). Overall, these data suggest that while E7820 resulted in RBM39 degradation and RNA mis-splicing in patients in vivo, the magnitude of these changes was far less than that achieved using in vitro treatment of preclinical models.

This is the first prospective clinical trial of an RBM39 degrader in patients with R/R, splicing factor mutant myeloid malignancies and the first trial to evaluate RBM39 degradation in humans. The safety profile of E7820 monotherapy was consistent with previously reported data [9]. Equally important, we provide the first evidence that RBM39 can be substantially degraded in patients with limited toxicity. Given prior preclinical data demonstrating that RBM39 degradation may enhance the activity of venetoclax in myeloid malignancies [15] as well as immune checkpoint blockade in solid tumors [7], the present data support future studies of E7820 in combination with these agents.

The limited clinical activity could be attributed to markedly less splicing disruption in patient samples compared to AML cell lines treated with E7820 doses exhibiting preclinical efficacy. It is unclear if this difference is due to pharmacodynamic limitations of RBM39 degradation in vivo at the dose administered or the fact that even greater RBM39 degradation may be required for maximal clinical activity. However, we used E7820 at the recommended phase 2 dose and higher doses of E7820 might be associated with prohibitive toxicity.

Although our trial was terminated early, our correlative studies showed that RBM39 degradation occurred early during treatment and additional cycles of treatment did not increase splicing disruption. This makes it unlikely that longer treatment would result in greater clinical efficacy.

In summary, our phase II trial of E7820 in patients with R/R, splicing factor-mutant myeloid malignancies provides proof-of-concept that splicing factor-mutant disease can be targeted in humans via RBM39 degradation. Despite limited clinical efficacy, the acceptable safety profile of E7820 and its ease of administration (oral daily dosing) would support its use in combination therapies.

### Supplementary information

Supplemental material

## Data Availability

Primary data are available from the corresponding author upon request (abdelwao@mskcc.org).
