# Supplementary material for: E7820, an anti-cancer sulfonamide, degrades RBM39 in patients with splicing factor mutant myeloid malignancies: a phase II clinical trial
Source: Leukemia. 2023 Oct 9;37(12):2512–6. doi: 10.1038/s41375-023-02050-4 (PMC10681888; doi:10.1038/s41375-023-02050-4)
Supplement: Supplementary file 1 — Supplemental material [file 41375_2023_2050_MOESM1_ESM.docx]

**- Supplement -**

**E7820, an Anti-Cancer Sulfonamide, Degrades RBM39 in Patients with Splicing Factor Mutant Myeloid Malignancies: A Phase II Clinical Trial**

Jan Philipp Bewersdorf^1,*^, Maximilian Stahl^2,*^, Justin Taylor^3^, Xiaoli Mi^1^, Namrata Sonia Chandhok^3^, Justin Watts^3^, Andriy Derkach^4^, Mateusz Wysocki^1^, Sydney X. Lu^5,6^, Jessie Bourcier^6^, Simon J. Hogg^6^, Jahan Rahman^6^, Sana Chaudhry^3^, Tulasigeri M Totiger^3^, Omar Abdel-Wahab^1,6,*^, and Eytan M. Stein^1,*^

1: Department of Medicine; Leukemia Service, Memorial Sloan Kettering Cancer Center, New York, NY

2: Department of Medical Oncology, Dana-Farber Cancer Institute, Boston, MA

3: Leukemia Program, Department of Medicine, University of Miami, Sylvester Comprehensive Cancer Center, Miami, FL

4: Department of Biostatistics and Epidemiology, Memorial Sloan Kettering Cancer Center, New York, NY

5: Myeloma Service, Department of Medicine, Memorial Sloan Kettering Cancer Center, New York, NY

6: Molecular Pharmacology Program, Sloan Kettering Institute, Memorial Sloan Kettering Cancer Center, New York, NY

*****These authors contributed equally.

**Corresponding authors:**

Omar Abdel-Wahab

Email: [abdelwao@mskcc.org](mailto:abdelwao@mskcc.org)

Phone: 347-821-1769

Eytan M. Stein

Email: [steine@mskcc.org](mailto:steine@mskcc.org)

Phone: 646-608-3749

**SUPPLEMENTARY MATERIALS AND METHODS**

*Patients*:

Adult (≥18 years) patients with R/R AML, MDS, or chronic myelomonocytic leukemia (CMML) with a hotspot splicing factor mutation in *SF3B1, SRSF2, U2AF1,* or *U2AF2* (with hotspot mutations as defined by OncoKB) or a nonsense or frameshift mutation in *ZRSR2* detected within 6 months of study screening were eligible for enrollment. Relapsed disease was defined as the reappearance of 5% or greater myeloblasts in the bone marrow or peripheral blood after achieving a complete remission (CR), CR with partial hematologic recovery (CRh), or CR with incomplete hematologic recovery (CRi) among AML patients or any relapse after achieving an International Working Group (IWG) defined response for MDS and CMML patients.(1, 2)

Refractory disease was defined as the failure to achieve an objective response after two cycles of intensive induction chemotherapy, two cycles of hypomethylating agent (HMA) plus venetoclax, or low-dose cytarabine plus glasdegib, or four cycles of HMA monotherapy. Patients were required to have adequate end organ function and an Eastern Cooperative Oncology Group (ECOG) performance status of ≤3. A full list or inclusion and exclusion criteria is provided in the **Supplemental Table 1**.

*Study design:*

This was an investigator-initiated, phase II trial (NCT05024994) conducted at Memorial Sloan Kettering Cancer Center and Sylvester Comprehensive Cancer Center at the University of Miami to explore the efficacy of E7820 in patients with R/R myeloid malignancies. Patients received 100 mg of E7820 daily during 28-day cycles until relapse, disease progression, development of unacceptable toxicity, allogeneic hematopoietic stem cell transplant (HSCT), or death. The dose of E7820 was based on the recommended phase II dose from solid tumor studies with dose modification permitted for adverse events as outlined in the study protocol.(3) If after six cycles of therapy AML and CMML patients did not achieve at least a partial remission (PR), and MDS patients did not at least achieve hematologic improvement (HI), study drug was discontinued. Participants who achieved an adequate response and were eligible for HSCT could proceed with HSCT after discontinuation of E7820. The study protocol was developed by the authors in collaboration with Eisai Pharmaceuticals and was approved by the individual Institutional Review Boards. All patients provided informed consent. The study was conducted in accordance with the Declaration of Helsinki.

*Definition of endpoints:*

The primary objective of this study was to evaluate the efficacy of E7820 in patients with R/R myeloid malignancies with mutations in splicing factor genes as measured by the overall response rate (ORR) within 6 cycles of therapy. The ORR was defined as a composite of CR + CRh for AML patients and as CR + PR for MDS and CMML patients. Response to treatment and treatment decisions in all participants was determined based on the 2017 ELN criteria for AML (1) and the International Working Group 2006 criteria for MDS (2) and 2015 for CMML.(4) Secondary objectives included an alternative definition of ORR (composite of CR, marrow CR, CRi, morphologic leukemia-free state [MLFS], PR, and HI), event-free survival (EFS) and 1-year OS rates. We assessed drug effects on RBM39 protein level, splicing of key target splicing events, global splicing events, changes in variant allele fraction (VAF) of splicing factor mutant clones, and DCAF15 mRNA levels and their correlation with clinical responses as exploratory, correlative endpoints. Toxicities were tabulated and graded according to the Common Terminology Criteria for Adverse Events Version 5 (CTCAE-5). Response assessment with bone marrow biopsies was performed at the end of cycle 1 and every 2 cycles thereafter.

*Patient mutational analysis:*

Mutational analysis of pre- and post-E7820 (from patient peripheral blood mononuclear cells (PB MNCs) or bone marrow mononuclear cells (BM MNCs) was performed using the MSK-IMPACT Heme(5) targeted next-generation sequencing platform as previously described.(5) This assay captures all coding regions of *SF3B1, SRSF2, U2AF1,* *U2AF2,* and *ZRSR2* as well as all genes known to be recurrently mutated in patients with myeloid malignancies.

*Western blot analysis of RBM39:*

Protein was harvested from patient peripheral blood mononuclear cells following Ficoll purification at indicated time points prior to therapy and on E7820 treatment. Lysate protein concentration was measured with the BCA reagent and 10-30 mcg was loaded per lane onto 4-12% NuPAGE^TM^ Bis-Tris protein gels. After transfer, PVDF membranes were probed with anti-RBM39 (Atlas Antibodies) and anti-actin (MilliporeSigma) antibodies at 1:1,000 and visualized by standard methods. Densitometry of protein bands were performed in ImageJ and normalized to actin for calculate relative RBM39 protein levels.

*RNA-sequencing library preparation and sequencing:*

For patient PBMNC and bone marrow mononuclear cell (BM MNC) RNA sequencing (RNA-seq), RNA was extracted using the Qiagen RNeasy extraction kit, according to the manufacturer’s instructions. For cell lines, RNA was extracted using similar methods from either K562 cells wild-type for *SF3B1/SRSF2* or with knockin of a single allele mutation in *SF3B1*^K700E^, *SF3B1*^K666N^, or *SRSF2*^P95H^ or NKM1 cells (which harbor a naturally occurring *U2AF1*^S34F^ mutation) after 24 hours of DMSO or E7820 (1μM) treatment. A minimum of 500 ng of high-quality RNA (as determined by Agilent Bioanalyzer) per replicate was used as input for library preparation. Poly(A)-selected, strand-specific (dUTP method) Illumina libraries were prepared by the Integrated Genomics Operation (IGO) at Memorial Sloan Kettering with a modified TruSeq protocol and sequenced on the Illumina HiSeq 2000 to obtain ∼100M 2x101 bp paired-end reads per sample.

*RNA sequencing analysis:*

Prior to mapping, raw FASTQ files were trimmed using Trim_galore (v0.6.4) to remove residual Illumina adapter and/or low quality (Q<15) sequences. Trimmed sequencing reads were then aligned to the human Hg19 reference genome (GENCODE, GRCh37.p13) using STAR (v2.7.5) (Dobin et al., 2013). Samtools (v1.9) was used to convert SAM files to BAM files, as well as sorting, and indexing. Sorted BAM files were used for read counting across genomic features (exons) with featureCounts (part of the subread package; v1.5.0) using the following parameters: -p -T 20 -O -F GTF -t exon.(6) The resultant counts file was used as input for differential gene expression analysis, which was performed using the edgeR (v3.32.1), DESeq2 (v1.30.1), and limma voom (v3.46.0) R packages.(7, 8) Data visualization and figure generation was performed in Rstudio (v1.3.1073) using the following packages: ggplot2 (v3.3.5) and complexHeatmap (v2.6.2). Read counts associated with genes were normalized using the median of ratios method to account for sequencing depth and RNA composition prior to comparison of DCAF15 expression across samples.

Differential alternative splicing events were detected using Multivariate Analysis of Transcript Splicing (rMATS, v4.1.1) using the GENCODE (v19) GTF annotation for GRCh37. rMATS was run with the –novelSS option so as to also identify novel, unannotated splice junctions not represented within the GTF annotation.(9-11) Enumeration of isoform counts was performed using only reads that span the splice junction directly. Pairwise sample comparisons were performed, and significant differential alternative splicing events were defined as those with greater than an absolute difference of 20% in isoform expression (|PSI|>0.2) and a false discovery rate of less than 5% (FDR<0.05). To identify robust AS events in all pairwise comparisons, junctions were first pre-filtered to remove those that possessed fewer than 30 reads on average. Shared and distinct splicing changes on E7820 treatment compared to baseline were identified in cell line, patient peripheral blood, and patient bone marrow samples with the same splicing factor mutation. Function and pathway enrichment was performed using Enrichr,(12-14) which includes GO Biological Process (2021), KEGG (2021), BioCarta (2016), Reactome (2022), MSigDB Hallmark (2020), and other gene-set libraries. All analyses were conducted within the R Programming environment with tools from Bioconductor.(15) The visualizations were created using the dplyr, tidyverse, ggplot2, ggvenn, and ComplexHeatmap packages.

*Sample size calculation:*

This study used an optimal Simon two-stage design. In the absence of an effective salvage therapy for patients with HMA failure and ORR of <10% with intensive chemotherapy or lower-intensity therapy, we used a null unpromising ORR of 10% and a promising rate of 30% to inform the sample size calculation. Per the study design 12 patients were enrolled in the first stage of the study. If no more than one patient achieved a response, the study was planned to close due to a lack of efficacy; otherwise, an additional 23 patients were planned to be accrued. If at the end of study, at least 6 of the 35 patients achieved a response, the study would be considered promising for further investigation. The type I and type II errors were both set at 0.10. As none of the first 12 patients enrolled achieved an objective response, the study was closed for futility.

*Statistical analysis:*

All patients who received at least one dose of study treatment were included in the efficacy evaluable population. Patients who discontinued treatment prior to the first response evaluation were considered as failure to achieve the primary response. ORR was estimated by sample proportion and confidence intervals were calculated based on exact binomial distribution. Kaplan-Meier methods were used to estimate the OS and EFS survival. OS was defined as the time from the first study dose to death. In the absence of death, patients were censored at the last follow up. EFS was defined as the time from the first study dose to relapse, death or primary refractory disease. If not response was achieved during course of the study, the time of the event would be day 1 of the trial. In the absence of death, refractory disease, and relapse, patients were censored at the last follow up. Exploratory endpoints were assessed on bone marrow biopsies collected at baseline, beginning of cycle 2, and at time of suspected response or progression. The change from baseline and the follow up time periods were summarized using summary statistics.

*Data Availability Statement:*

RNA-seq data generated in the study has been uploaded to the NIH’s Gene Expression Omnibus (GEO) under the accession number GSE227343.

**Supplemental Figures**

**Supplemental Figure 1. Oncoprint of baseline mutations in the E7820 phase II clinical trial cohort.** Oncoprint of baseline mutations and clinical characteristics of patients enrolled in the E7820 phase II clinical trial cohort.

**
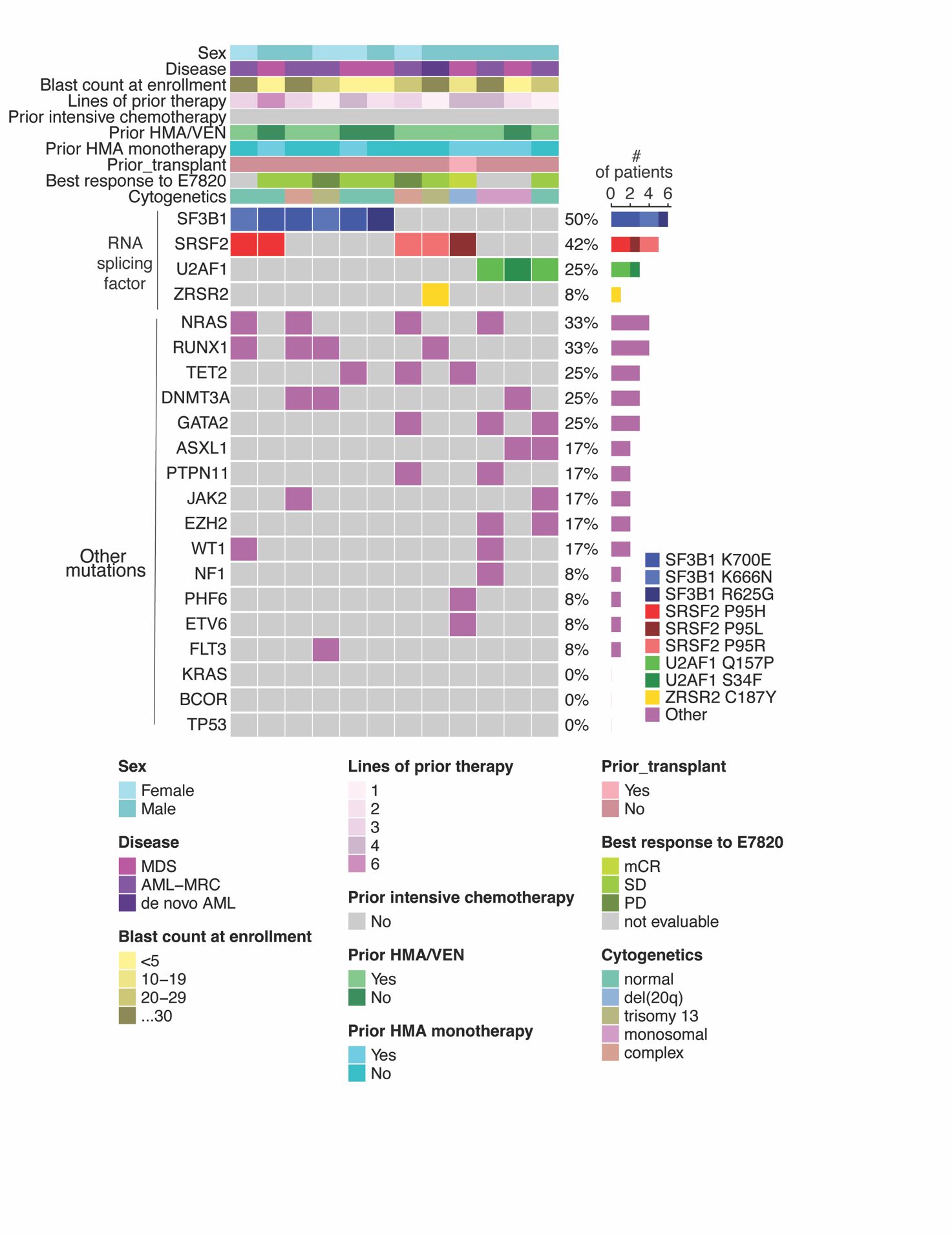
**

**Supplemental Figure 2: Variant allele frequency of mutations in RNA splicing factors at study enrollment versus on treatment with E7820.** Shown are the individual VAF values for each patient and the box-and-whisker plot represents the median (middle) and maximum and minimum values (top and bottom whisker respectively). Mutant allele frequency was determined by clinical MSK IMPACT targeted gene sequencing of peripheral blood and/or bone marrow.

**
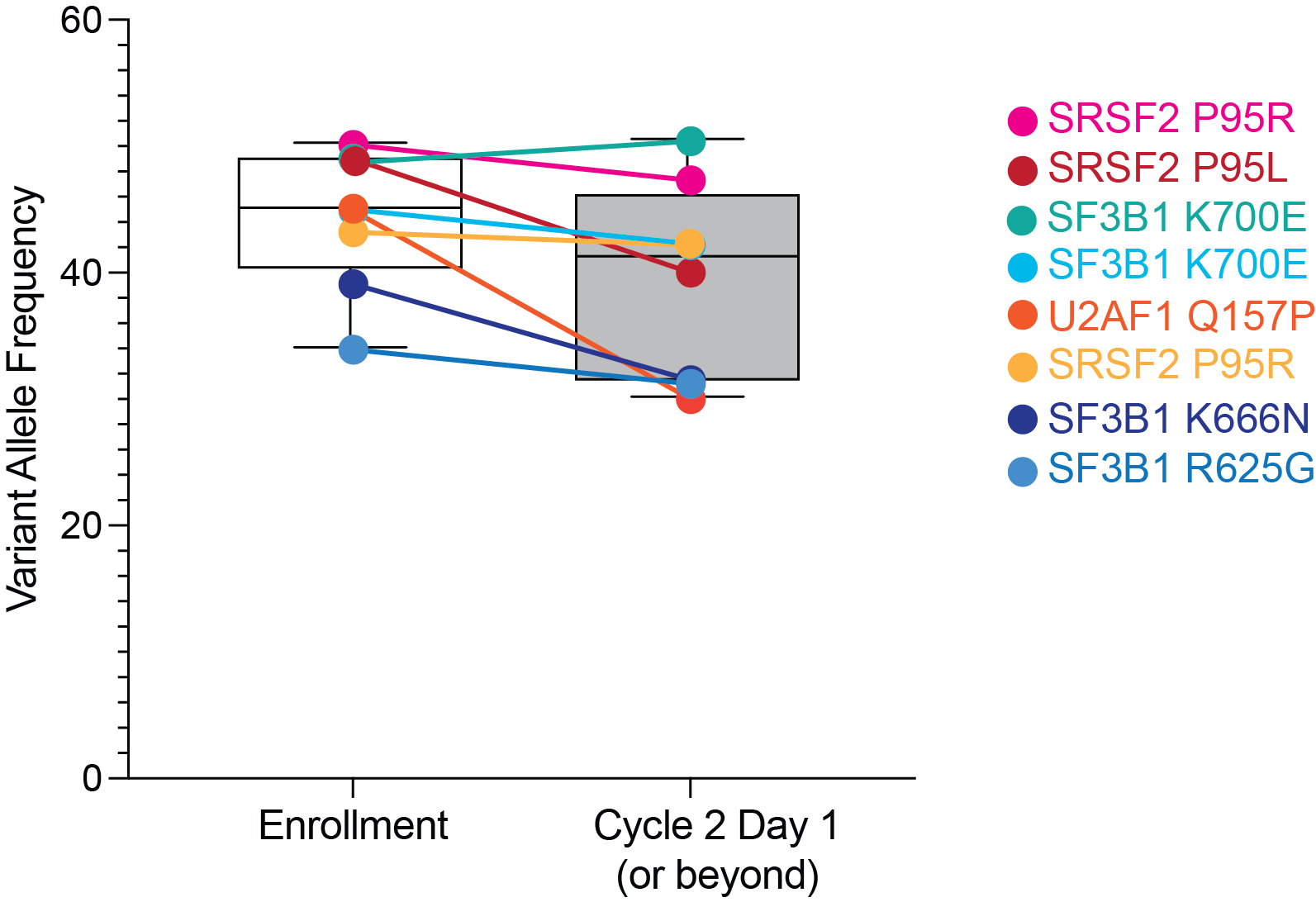
**

**Supplemental Figure 3: Overall survival of patients treated with E7820.** Kaplan-Meier curve of overall survival (OS) for patients from the date of E7820 initiation to death or last follow up. At a median duration of follow up of 13.1 months (95% confidence interval [95% CI]: 10.3 months – not reached), the median OS from the time of E7820 initiation to death was 3.8 months (95% CI: 1.5 months – not reached).

**Supplemental Figure 4: Stable expression of DCAF15 pre-tretament versus on treatment with E7820.** Data are from RNA sequencing of cell lines (in presence of DMSO or E7820 treatment) as well as bone marrow (BM) and peripheral blood from patient samples pre-treatment or on-treatment with E7820 from the phase II trial.

**
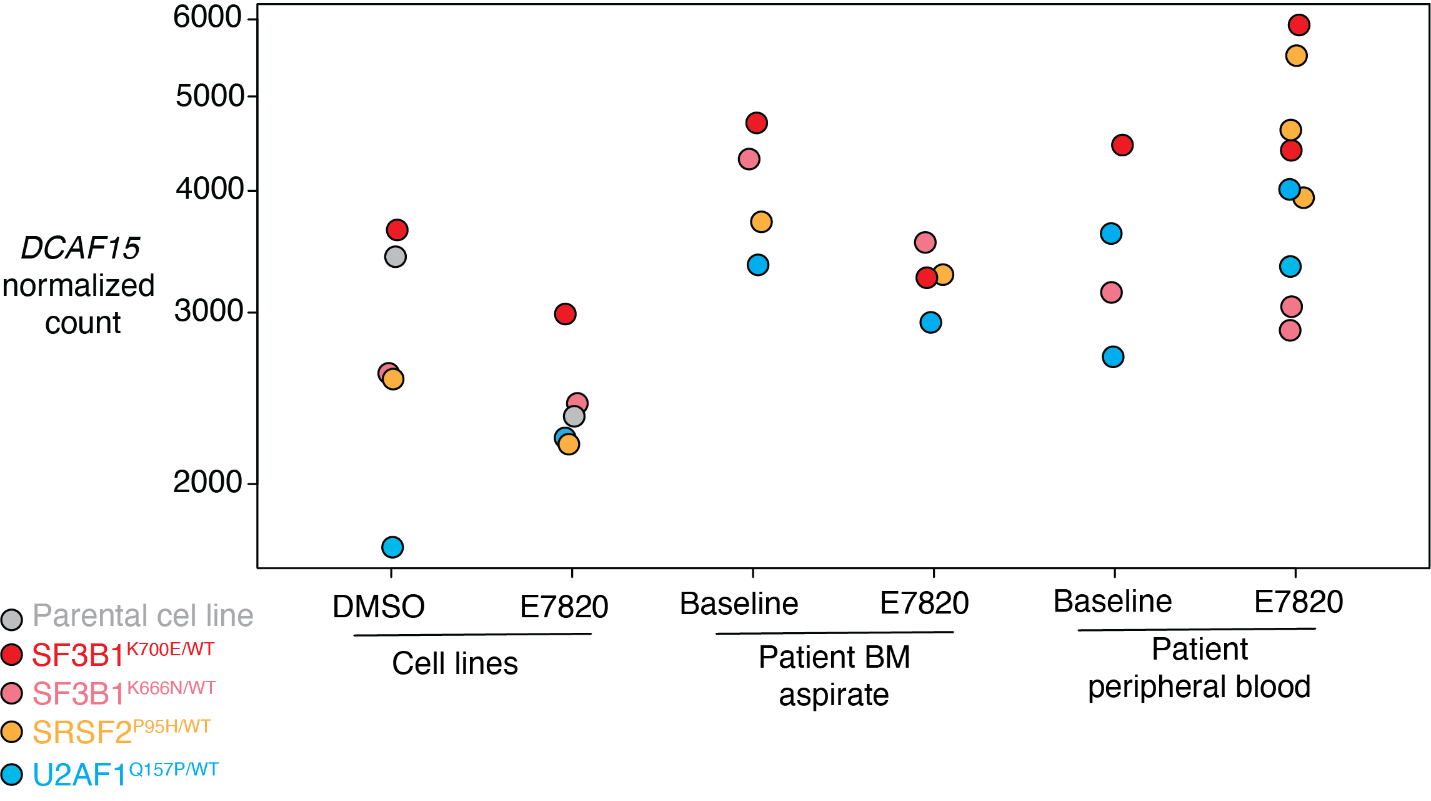
**

**Supplemental Figure 5: Principle component analyses and hierarchical clustering of gene expression in cell lines and patient samples in presence or absence of E7820 treatment from RNA-seq. (A)** PCA analyses of gene expression in the cell lines and tissues evaluate by RNA-seq. Abbreviations- BMA: bone marrow aspirate; PB: peripheral blood. **(B)**

Hierarchical clustering of gene expression in the same samples from (A). As shown cell lines and patient samples clustered differently regardless of splicing factor mutation or drug treatment despite being processed and sequenced altogether.

**
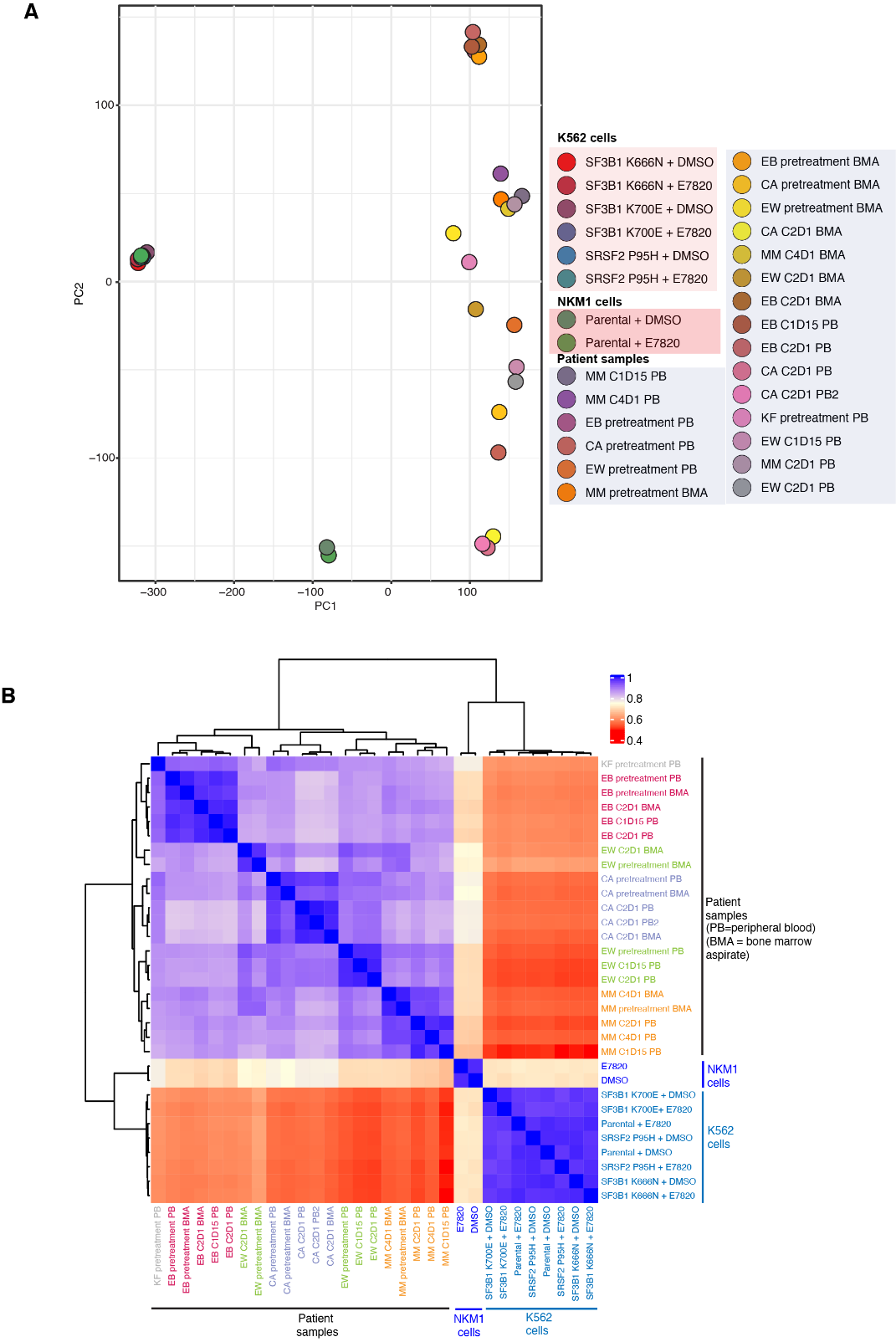
**

**Supplemental Figure 6: Modest overlap in differnetially spliced transcripts between AML patient samples and preclinical samples upon E7820 treatment. (A)** Venn diagram of significant differential alternative splicing events in DMSO versus E7820-treated K562 cells harboring *SF3B1*^K666N^ as well as in PB and BM of a patient with *SF3B1*^K666N^. Gene ontology (GO) enrichment analysis of differentially spliced RBM39-responsive events **(B)** unique to the cell line or **(C)** common across cell lines and patient samples.

**
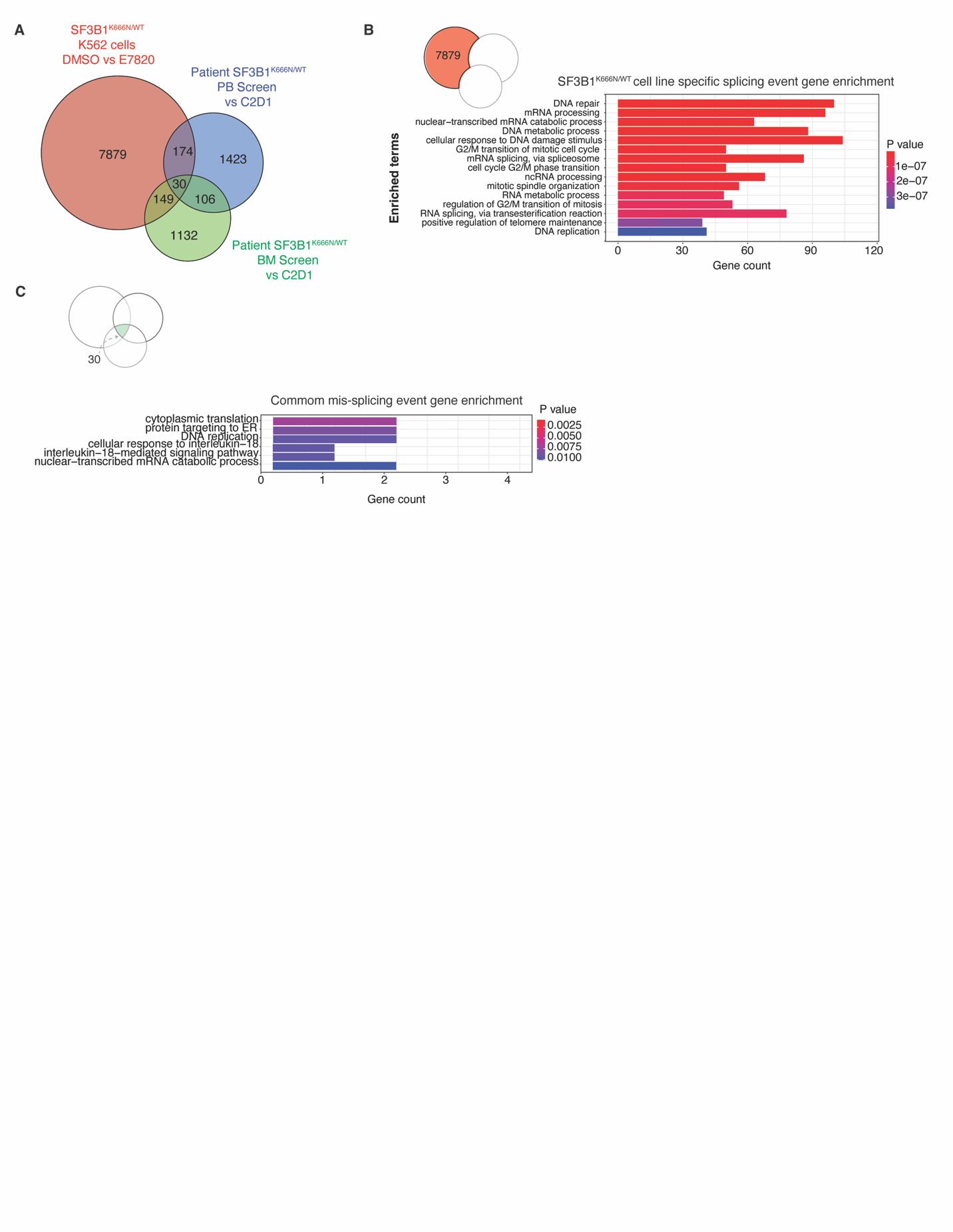
**

**Supplemental Table 1: Overview of key inclusion and exclusion criteria.**

| **Inclusion criteria** | **Exclusion criteria** |
| --- | --- |
| Subject is ≥ 18 years of age at the time of signing informed consent | Patients with acute promyelocytic leukemia |
| Subject has relapsed or refractory MDS, AML or CMML with a previously defined hotspot splicing factor mutation in *SF3B1, SRSF2, U2AF1* or *U2AF2* (with hotspot mutations as defined by OncoKB) or a nonsense or frameshift mutation in *ZRSR2*. A splicing factor mutation is required to be detected on next generation sequencing from bone marrow aspirate or peripheral blood at any timepoint within the 6 months prior to screening for the study.   1. Relapsed AML is defined as the appearance of 5% or greater myeloblasts in the bone marrow or peripheral blood after achieving a CR (MRD positive or negative), CRh, or CRi. Patients with mutations in FLT3, IDH1 or IDH2 must have failed or been intolerant of an FDA approved FLT3, IDH1 or IDH2 inhibitor before enrolling on study. 2. Refractory AML is defined as failure to achieve a CR, CRh, or CRi after one of the following regimens:    1. Two cycles of intensive induction chemotherapy with a cytarabine containing regimen (e.g. 7+3, MEC, HIDAC, etc.)    2. Two cycles of HMA/venetoclax or LDAC/glasdegib    3. 4 cycles of HMA monotherapy 3. Relapsed MDS is defined as any relapse after achieving an IWG defined response. 4. Refractory MDS is defined as:    1. For patients with intermediate, high or very high risk disease by IPSS-R, the failure to achieve a response (as per IWG 2006 criteria) after 4 cycles of HMA monotherapy or 2 cycles of HMA + venetoclax.    2. For patients with very low and low risk disease by IPSS-R failure to achieve hematologic improvement or loss of hematologic improvement after treatment with standard of care agents such as ESAs, Luspatercept (for MDS with ringed sideroblasts) and lenalidomide (for pts with a 5q-). 5. e. Relapsed CMML is defined as any relapse after achieving an IWG defined response. 6. f. Refractory CMML is defined as failure to achieve a response (as per IWG 2006 criteria) after 4 cycles of HMA monotherapy or 2 cycles of HMA + venetoclax. | Subject has immediate life-threatening, severe complications of their myeloid malignancy such as uncontrolled bleeding, pneumonia with hypoxia or shock, and/or disseminated intravascular coagulation |
| Subject has an Eastern Cooperative Oncology Group (ECOG) performance status of 0-3 | Subject has significant active cardiac disease within 6 months prior to the start of study treatment, including New York Heart Association (NYHA) class III or IV congestive heart failure; acute coronary syndrome (ACS); and/or stroke or left ventricular ejection fraction (LVEF) <40% by echocardiogram (ECHO) or multi-gated acquisition (MUGA) scan obtained within 28 days prior to the start of study treatment. |
| Subject has adequate organ function defined as:   - Serum aspartate aminotransferase/serum glutamic oxaloacetic transaminase (AST/SGOT) and alanine aminotransferase (ALT/SGPT) ≤ 3 x ULN, unless considered due to organ involvement by the patient’s myeloid malignancy (in that case a cut off of ≤ 5 x ULN will be used) - Serum direct bilirubin < 1.5 x ULN. - Creatinine clearance ≥ 60 mL/min based on the Cockroft-Gault glomerular filtration rate (GFR) estimation. - Females of childbearing potential may participate provided they have a negative serum pregnancy test at screening and a negative serum OR urine pregnancy test within 72 hours of starting on treatment. Females and male participants with female partners of childbearing potential also must agree to either abstain from sexual intercourse or use a highly effective method of contraception while on study and for 4 months after completing the study treatment. - In case of leukemic organ involvement, patients with creatinine clearance > 30 ml/min and bilirubin ≤ 2.0 x ULN will be eligible to be included. | Subject has active viral infection with human immunodeficiency virus (HIV), or active infection with hepatitis B virus (HBV) or hepatitis C virus (HCV). Patients with HIV that is controlled with anti-retroviral therapy are eligible to participate. |
| There are no limits on transfusion and/or growth factor support for enrollment. | Subject is known to have dysphagia, short-gut syndrome, gastroparesis, or other conditions that limit the ingestion or gastrointestinal absorption of drugs administered orally. |
|  | Subject has active uncontrolled systemic fungal, bacterial, or viral infection (defined as ongoing signs/symptoms related to the infection without improvement despite appropriate antibiotics, antiviral therapy, and/or other treatment). |
|  | Subject has QTc interval (i.e., Fridericia’s correction [QTcF]) ≥ 480 ms or other factors that increase the risk of QT prolongation or arrhythmic events (e.g., heart failure, family history of long QT interval syndrome) at screening. Patients with left bundle branch block or right bundle branch block with prolonged QTc will be allowed to enroll on the trial with medical monitor approval. |
|  | Female subject who is pregnant or lactating. |
|  | Subject with known hypersensitivity to sulfa medications |

**Supplemental Table 2: Baseline patient, disease, and clinical characteristics of patients included in Figure 1A.**

| **Patient** | **Disease** | **Prior therapies** | **Best response to E7820** | **Splicing mutation** | **Other concurrent genetic alterations** | **Karyotype** |
| --- | --- | --- | --- | --- | --- | --- |
| Patient #1 | AML-MRC | Decitabine + venetoclax | Stable disease | *U2AF1* Q157P | *ASXL1, JAK2, EZH2, GATA2* | 46,XY[20] |
| Patient #2 | MDS-EB2 | Azacitidine + venetoclax -> allo-HCT -> azacitidine-> PRMT5 inhibitor | Marrow Complete Remission | *SRSF2* P95L | *TET2, SAMHD1, SRC, TOP1, PLCG1, PTPN1, SH2B3, ATXN2, ARID2, ETV6, PHF6, RAD21* | 46,XY,del(20)(q11.2q13.1)[20] |

**Supplemental Table 3: Adverse events (>10% incidence rate).**

| **Adverse event** | **Any grade (n, %)** | **≥ Grade 3 (n, %)** |
| --- | --- | --- |
| **Hematologic adverse events** | | |
| Anemia | 2 (16.7%) | 2 (16.7%) |
| Neutropenia | 2 (16.7%) | 2 (16.7%) |
| **Non-hematologic adverse events** | | |
| Diarrhea | 6 (50.0%) | 0 |
| Cough | 4 (33.3%) | 0 |
| Non-cardiac chest pain | 3 (25.0%) | 0 |
| Epistaxis | 3 (25.0%) | 1 (8.3%) |
| Fall | 3 (25.0%) | 1 (8.3%) |
| Nausea | 3 (25.0%) | 0 |
| Abdominal pain | 2 (16.7%) | 0 |
| Fever | 2 (16.7%) | 0 |
| Hematoma | 2 (16.7%) | 2 (16.7%) |
| Lung infection | 2 (16.7%) | 2 (16.7%) |
| Dizziness | 2 (16.7%) | 0 |
| Fatigue | 2 (16.7%) | 0 |
| Flatulence | 2 (16.7%) | 0 |
| Respiratory failure | 2 (16.7%) | 2 (16.7%) |
| Sepsis | 2 (16.7%) | 2 (16.7%) |
| Pain | 2 (16.7%) | 0 |

**Supplemental Table 4: Baseline patient, disease, and clinical characteristics of patients included in Figure 1B-E.**

| **Patient** | **Disease** | **Prior therapies** | **Best response to E7820** | **Splicing mutation** | **Other concurrent genetic alterations** | **Karyotype** |
| --- | --- | --- | --- | --- | --- | --- |
| Patient #1 | MDS-RS-MLD | Azacitidine -> Decitabine ->  Luspatercept ->  Lenalidomide | Stable disease | *SF3B1* K700E | *TET2, ESR1, SH2B3* | 46,XX [20] |
| Patient #2 | AML-MRC | Azacitidine + venetoclax -> decitabine + venetoclax | Progressive disease | *SF3B1* K666N | *DNMT3A, RUNX1, ASXL2, EED, FLT3,* | 47,XX,+13[4]/46,XX [16] |
| Patient #3 | AML-MRC | Decitabine + venetoclax | Stable disease | *U2AF1* Q157P | *ASXL1, JAK2, EZH2, GATA2* | 46,XY [20] |
| Patient #4 | MDS-EB2 | Azacitidine + venetoclax -> allo-HCT -> azacitidine-> PRMT5 inhibitor | Marrow Complete Remission | *SRSF2* P95L | *TET2, SAMHD1, SRC, TOP1, PLCG1, PTPN1, SH2B3, ATXN2, ARID2, ETV6, PHF6, RAD21* | 46,XY,del(20)(q11.2q13.1) [20] |

**References:**

1. Dohner H, Estey E, Grimwade D, Amadori S, Appelbaum FR, Buchner T, et al. Diagnosis and management of AML in adults: 2017 ELN recommendations from an international expert panel. Blood. 2017;129:424-47.

2. Cheson BD, Greenberg PL, Bennett JM, Lowenberg B, Wijermans PW, Nimer SD, et al. Clinical application and proposal for modification of the International Working Group (IWG) response criteria in myelodysplasia. Blood. 2006;108:419-25.

3. Mita M, Kelly KR, Mita A, Ricart AD, Romero O, Tolcher A, et al. Phase I study of E7820, an oral inhibitor of integrin alpha-2 expression with antiangiogenic properties, in patients with advanced malignancies. Clin Cancer Res. 2011;17:193-200.

4. Savona MR, Malcovati L, Komrokji R, Tiu RV, Mughal TI, Orazi A, et al. An international consortium proposal of uniform response criteria for myelodysplastic/myeloproliferative neoplasms (MDS/MPN) in adults. Blood. 2015;125:1857-65.

5. Durham BH, Getta B, Dietrich S, Taylor J, Won H, Bogenberger JM, et al. Genomic analysis of hairy cell leukemia identifies novel recurrent genetic alterations. Blood. 2017;130:1644-8.

6. Liao Y, Smyth GK, Shi W. featureCounts: an efficient general purpose program for assigning sequence reads to genomic features. Bioinformatics. 2014;30:923-30.

7. Law CW, Chen Y, Shi W, Smyth GK. voom: precision weights unlock linear model analysis tools for RNA-seq read counts. Genome Biol. 2014;15:R29.

8. Love MI, Huber W, Anders S. Moderated estimation of fold change and dispersion for RNA-seq data with DESeq2. Genome Biol. 2014;15:550.

9. Shen S, Park JW, Lu ZX, Lin L, Henry MD, Wu YN, et al. rMATS: robust and flexible detection of differential alternative splicing from replicate RNA-Seq data. Proc Natl Acad Sci U S A. 2014;111:E5593-601.

10. Park JW, Tokheim C, Shen S, Xing Y. Identifying differential alternative splicing events from RNA sequencing data using RNASeq-MATS. Methods Mol Biol. 2013;1038:171-9.

11. Shen S, Park JW, Huang J, Dittmar KA, Lu ZX, Zhou Q, et al. MATS: a Bayesian framework for flexible detection of differential alternative splicing from RNA-Seq data. Nucleic Acids Res. 2012;40:e61.

12. Chen EY, Tan CM, Kou Y, Duan Q, Wang Z, Meirelles GV, et al. Enrichr: interactive and collaborative HTML5 gene list enrichment analysis tool. BMC Bioinformatics. 2013;14:128.

13. Kuleshov MV, Jones MR, Rouillard AD, Fernandez NF, Duan Q, Wang Z, et al. Enrichr: a comprehensive gene set enrichment analysis web server 2016 update. Nucleic Acids Res. 2016;44:W90-7.

14. Xie Z, Bailey A, Kuleshov MV, Clarke DJB, Evangelista JE, Jenkins SL, et al. Gene Set Knowledge Discovery with Enrichr. Curr Protoc. 2021;1:e90.

15. Huber W, Carey VJ, Gentleman R, Anders S, Carlson M, Carvalho BS, et al. Orchestrating high-throughput genomic analysis with Bioconductor. Nat Methods. 2015;12:115-21.
